# Clinical Utility of the Pathogenesis-Related Proteins in Alzheimer’s Disease

**DOI:** 10.3390/ijms21228661

**Published:** 2020-11-17

**Authors:** Bin Zhou, Masanori Fukushima

**Affiliations:** 1 Division of Medical Innovation, Translational Research Center for Medical Innovation, Foundation for Biomedical Research and Innovation, Kobe 650-0047, Hougo, Japan; 2 Medical R&D, Fukushima & Partners, Nagoya 458-0045, Aichi, Japan; mfukushi19481130@docomo.ne.jp

**Keywords:** Alzheimer’s disease, biomarkers dynamics, interaction, time order

## Abstract

Research on the Aβ cascade and alternations of biomarkers in neuro-inflammation, synaptic dysfunction, and neuronal injury followed by Aβ have progressed. But the question is how to use the biomarkers. Here, we examine the evidence and pathogenic implications of protein interactions and the time order of alternation. After the deposition of Aβ, the change of tau, neurofilament light chain (NFL), and neurogranin (Ng) is the main alternation and connection to others. Neuro-inflammation, synaptic dysfunction, and neuronal injury function is exhibited prior to the structural and metabolic changes in the brain following Aβ deposition. The time order of such biomarkers compared to the tau protein is not clear. Despite the close relationship between biomarkers and plaque Aβ deposition, several factors favor one or the other. There is an interaction between some proteins that can predict the brain amyloid burden. The Aβ cascade hypothesis could be the pathway, but not all subjects suffer from Alzheimer’s disease (AD) within a long follow-up, even with very elevated Aβ. The interaction of biomarkers and the time order of change require further research to identify the right subjects and right molecular target for precision medicine therapies.

## 1. Introduction

The amyloid hypothesis of Alzheimer’s disease (AD) proposes that accumulation of amyloid-beta (Aβ) in the brain triggers pathogenesis of AD and the cascade of the spread of tau-related neurofibrillary tangles, neuro-inflammation, and neuronal degeneration. The failure of the anti-Aβ clinical trials indicated that the therapeutic potential of BACE-1(beta-site APP cleaving enzyme1) inhibition and anti-amyloid antibody is doubling [1,2,3,4]. The question of the amyloid hypothesis in Alzheimer’s disease has been raised in recent years.

However, much evidence still confirms that Aβ appears to be the initial disease mechanism in AD. Among initially amyloid-negative adults, Farrell found a regionally specific association between declining episodic memory and increased amyloid accumulation across multiple posterior cortical regions [5,6].

In the event of failure of clinical trials, Knopman [1] considered the selection of a population with positive Aβ to be too late for a maximum treatment effect. Bischol [7] and Palmqvist [6] have proposed a potential solution for enrolling subjects with threshold amyloidosis. From experience, defining the threshold is very difficult. A higher SUVR (standard uptake value ratio) of Aβ does not always indicate a late AD stage. At the same level of Aβ positron emission tomography-computed tomography (PET), higher 18F-positron emission tomography-computed tomography (FDG-PET) reduces the risk of AD in MCI (mild cognitive impairment) for normal subjects. Even in subjects with very high SUVR of Aβ, the modulation of higher FDG-PET continues to reduce the risk of conversion to AD [8].

Many studies have shown that in the downstream of Aβ, proteins have changed the spread of tau-related neurofibrillary tangles, synaptic dysfunction, neuro-inflammation, neuronal injury, and neuronal degeneration, all of which are associated with the pathogenesis of AD [6,7,9]. However, the interaction and the time order of changes of the proteins are not clear yet, which makes it difficult to clarify the molecular pathway. Palmqvist [6] proposed one order of the biomarkers based on the accumulation of Aβ PET in a cross-sectional data that could not reflect the time order. As noted by the author, it requires the follow-up of Aβ PET and the rates of change of Aβ.

In the hypothesis of AD pathogenesis proposed by Jack [10], the change of MRI and PDG-PET is accompanied by the accumulation of Aβ. Some studies showed that accumulation of Aβ resulted in hippocampal atrophy. However, the deposition of Aβ does not imply that the hippocampus is definitely atrophied. Aβ pathology and hippocampal atrophy are independently associated with memory function in cognitively healthy elderly people [11]. In the prediction analysis, MCI or normal cognition subjects with positive Aβ and hippocampal atrophy an increased risk of transition to AD. We also observed that not all subjects with elevated Aβ have hippocampal atrophy. The change in glucose metabolism measured by FDG-PET showed similar results. The involvement of structural MRI and glucose metabolism measured by FDG-PET with the pathogenesis of AD can be independent of Aβ. Successful treatment strategies can be devised by understanding the contribution of these markers to different aspects of disease pathogenesis.

**Figure 1:**
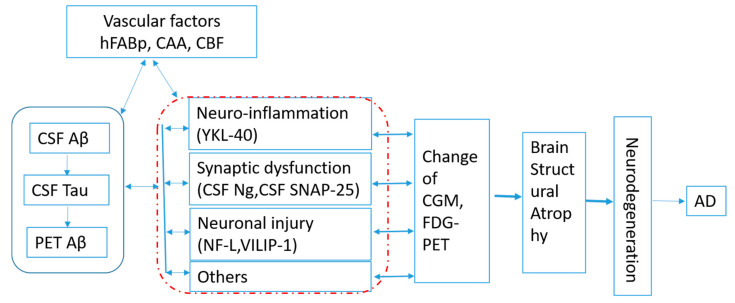
Hypothesis of Alzheimer’s disease (AD) pathogenesis. HFABp: heart-type fatty acid binding protein, CAA: cerebral amyloid angiopathy, CBF: cerebral blood flow, CGM: cerebral glucose metabolism, FDG-PET: 18F-positron emission tomography-computed tomography, CSF Aβ: cerebral spinal fluid beta amyloid, CSF Tau: cerebral spinal fluid Tau protein, PET-Aβ: Amyloid-beta assessed by florbetapir PET. Ng: neurogranin; SNAP-25: synaptosomal-associated protein 25; YKL-40: chitinase-3-like protein 1; NFL: neurofilament light chain; VILIP-1:visinin-like protein 1.

The framework of amyloid (A), tau (T), and neurodegeneration (N) biomarkers is proposed to define the state of patients with regard to Alzheimer’s-related pathologic change. The group with abnormal levels of amyloid, tau, and neurodegeneration (A+T+N+) showed consistently greater cognitive decline than the group with normal levels of all biomarkers (A−T−N−) [12]. The ATN system has different implications in patients with vs. without dementia and poses a challenge to discriminate between patient populations with specific features [13] and in terms of clinical trial design. Timing is always the challenge for subject enrollment in AD clinical trials [1,14]. Kant [15] proposed Apolipoprotein E, the endocytic system, and microglial activation as regulators of tau pathology, which can progress independently of Abeta accumulation. Panza [16] suggested alternative strategies, including interference with modifiable risk factors. Figuring out the pathway that includes pathological molecular changes and related association can help us clearly define the timing and identify the modifiable factors in both Abeta and tau progress for precision therapeutics. Based on the published results, efforts shall be made to clarify the interactions and time order of the protein changes in order to set up the pathway of AD pathogenesis, as shown in Figure 1.

## 2. The Change of Biomarkers in Alzheimer’s Disease

### 2.1. Aβ and Tau

The changes and association among the biomarkers in AD are showed in Table 1. Research indicates that cerebral spinal fluid (CSF) Aβ decreased while brain PET Aβ increased in AD when compared with CN (cognitive normal). CSF t-Tau and CSF p-tau increased at an early stage of AD pathogenesis compared to the mild cognitive impairment (MCI) and normal cognition groups [17,18,19]. Longitudinally, CSF Aβ decreased in all groups; however, t-tau and p-tau levels increased significantly in the CN+ or CN group, while it decreased in the AD+ or AD group [17,18].

### 2.2. Synaptic Dysfunction

Synaptic dysfunction and degeneration are early fundamental pathophysiological characteristics of AD. Biomarkers that can track synaptic dysfunction in AD are eagerly awaited. The major proteins of synaptic dysfunction involved in the pathogenesis of AD are neurogranin (Ng), synaptosomal-associated protein 25 kDa (SNAP-25), soluble N-ethylmaleimide-sensitive factor attachment protein receptor (SNARE complex), synaptotagmin, syntaxin, Ca2+, complexins-I/II (CPLX1/2) (GABAergic/glutamatergic selectively expressed proteins). Syntaxin-1, SNAP25, and VAMP are three SNARE proteins.

Ng is a calmodulin-binding protein expressed primarily in the brain, particularly in dendritic spines, and participating in the protein kinase C signaling pathway. Ng is the main postsynaptic protein binding and regulating calmodulin in the absence of calcium. CSF Ng is increased in AD compared to CN or Parkinson’s disease frontotemporal dementia and amyotrophic lateral sclerosis [19,20,21,22]. Baseline levels of Ng were significantly higher in the AD+ group than the CN and MCI− groups. The levels were also higher in the MCI+ group compared to the Aβ− (CN and MCI) groups. Longitudinally, CSF Ng or brain Ng significantly decreased in the AD+ group or AD patients [15,16]. Ng/BACE1 levels were elevated in both subjective cognitive decline and mild cognitive impairment compared to healthy controls [23] and can distinguish between depression and AD among patients with similar cognitive deficits, along with the classic AD biomarkers [24].

SNAP-25 is one of the key proteins involved in the formation of the SNARE complex, which is responsible for the calcium-dependent exocytosis of neurotransmitters and the key to the normal functioning of the brain. SNAP-25 increased in AD or MCI versus CN [14,19,22]. However, Beeri [42] and Shimohama [58] indicated dementia to be associated with reductions in SNAP-25. Longitudinally, SNAP-25 levels declined significantly in the AD+ group, which indicates varying level of SNAP-25 at different stages of AD [17]. The levels of SNAP-25 in serum carried by neuron-derived exosomes (NDEs) were reduced in AD patients compared to healthy controls [59]

Neuronal Pentraxin 2 (NPTX2) were decreased, and Aβ1-42/Tau or NPTX2/Tau discriminated AD and controls best. NPTX2/Tau correlated strongly with cognition in AD and MCI and predicted a 2–3-year decline [19]. 

In AD, the levels of synaptobrevin and synaptophysin decreased significantly from the levels of control. Other synaptic factors of synaptotagmin, syntaxin 1/HPC-1, complexin-1, complexin-2, and septin-5 have also been reported to increase [22,24,42].

### 2.3. Neuroinflammation

The inflammation associated proteins include microglial function, TREM2 (triggering receptor expressed on myeloid cell 2), YKL-40, IP-10, complement C, etc.

Chitinase-3-like protein 1 (CHI3L1), also known as YKL-40, is a secreted glycoprotein of 40 kDa in size and is encoded by the CHI3L1 gene in humans, a gene localized on chromosome 1q31-q32, and the crystal structure of YKL-40 has been described [60]. YKL-40 was initially associated with the macrophage lineage [61]. During neuroinflammatory processes, its expression is abundant in reactive astrocytes and residual in microglial cells [49]. In the brain, YKL-40 is upregulated in pulmonary disease and several neurological disorders such as stroke, lentiviral encephalitis, traumatic brain injury, and multiple sclerosis [62,63,64,65]

Elevated concentrations of CSF YKL-40, monocyte chemotactic protein-1 (MCP-1), visinin-like protein 1 (VILIP-1), and soluble variant TREM2 (sTREM2) in CSF were observed in AD when compared with the control in a meta-analysis [66,67]. Baseline YKL-40 was significantly higher in the AD+ group when compared with the MCI− group. Longitudinally, YKL-40 in all Aβ+ and Aβ- groups of AD, MCI, and CN showed an increase in mean levels over time [17]. Not only occurring in AD, high YKL-40 in CSF is associated with FTLD (frontotemporal lobar degeneration) [67]. Using post-mortem humans, Singh-Bains [43] demonstrated that there was a 91% and 69% increase in the expression and load of Iba1 (ionized calcium-binding adapter molecule, microglia), respectively; the process length and branching of HLA-DR (microglia) positive cells was reduced by 33% and 49%, respectively; there was a 27% increase in the astrocytes (GFAP, fibrillary acidic protein) basement-membrane associated molecules. Fibronectin expression in AD. Microglial and neurovascular dysfunction act as drivers of AD. In the case of TREM2, plasma sTREM2 does not differ between healthy controls, mild cognitive impairment (MCI), or AD [68].

### 2.4. Neuronal Injury

Cerebrospinal fluid (CSF) neuro-filament light (NFL) is a protein biomarker of axonal injury and axonal degeneration. Visinin-like protein 1 (VILIP-1) is a protein encoded by the VSNL1 gene in humans. This gene is a member of the visinin/recoverin subfamily of neuronal calcium sensor proteins. NfL and VILIP-1 of neuronal injury have been reported to predict more frontotemporal dementia (FTD) disease progression than AD.

Synaptic damage, axonal neurodegeneration, and neuroinflammation are common features in Alzheimer’s disease (AD), frontotemporal dementia (FTD), and Creutzfeldt–Jakob disease (CJD). Although there was a stepwise increase in CSF NfL levels between control participants, participants with MCI, and Alzheimer’s disease, the concentrations of NfL were highest in participants with amyotrophic lateral sclerosis (ALS) and frontotemporal dementia (FTD) [69]. Research indicated that neuronal injury-related biomarkers of YKL-40 and NfL are valuable tools for staging and predicting patients within the ALS/FTD clinical spectrum [70], as a disease progression biomarker in genetic frontotemporal dementia [45,71], or in distinguishing behavioral variant frontotemporal dementia (bvFTD) from primary psychiatric disorders (PSY) [72].

NfL has the potential to assist in the differentiation of FTD from AD and PD from atypical parkinsonian syndromes [73]. NfL is not a specific biomarker for the diagnosis or progression of AD.

VILIP-1, a marker of neuronal injury, reflects functional and structural changes in AD brains. VILIP-1 levels are significantly higher in AD compared to normal controls [74,75]. As CSF VILIP-1 is an unspecific marker for neuronal injury and CSF myelin basic protein reflects neuroaxonal demyelination, the results of longer periods of stress associated with higher levels of CSF VILIP-1 suggested that long-term stress may be associated with neurodegenerative processes in the brain [76].

The correlations between microglial activation and tau or amyloid deposition were stronger in Alzheimer’s disease than in mild cognitive impairment, suggesting that these pathologies increase together as the disease progresses [77]. 

### 2.5. Vascular Factors

Research has shown that cerebrovascular processes are involved in the onset of the pathogenesis of AD, in which cerebral amyloid angiopathy (CAA), cerebral blood flow (CBF), and pericytes are of the main factors. Accumulating evidence indicates that the intersections between CAA and AD has a crucial role to play in improving vascular function in the treatment of both diseases and indicated the next steps needed to identify therapies [78]. Cilostazol, a selective inhibitor of phosphodiesterase (PDE) III promoted perivascular drainage of soluble fluorescent Aβ1-40 in Tg-SwDI mice [26]. However, the clinical trial of Cilostazol on the prevention of MCI is ongoing [79]. Atrial fibrillation (AF) has an increased dementia risk [25,28]. Angiotensin receptor blockers (ARBs) were associated with lower risk of dementia for all patients [28]. A heathy blood–brain barrier (BBB) protects neurons to maintain the proper synaptic and neuronal functioning. BBB breakdown leads to neuronal injury, synaptic dysfunction, loss of neuronal connectivity [29]. Pericyte deterioration may cause BBB breakdown and white matter attenuation [30], capillary constriction and CBF reduction [34,80], and clearance of Abeta [32]. The decrease of CBF and white matter attenuation are associated with AD pathology [27,35,81]. 

## 3. The Interaction between the Proteins and Association with Neuroimaging Findings 

### 3.1. Interaction between the Proteins

Based on the Aβ cascade hypothesis, the deposition of Aβ results in the change of the biomarkers. Beyond the levels of individual proteins, protein–protein interactions that are critical to the process of vesicular neurotransmission also contribute [36]. Identifying protein interactions may make it easier to understand the pathogenesis and early prevention of AD.

55% of MCI and 83% of AD subjects had a high Aβ load [25]. Baseline CSF t-Tau levels were significantly elevated in the AD+ group when compared with Aβ+ (MCI and CN) and Aβ- (MCI and CN) groups. Longitudinally, t-Tau levels significantly increased in both the CN and MCI+ groups but decreased in the AD+ group. [17].

The synaptic CSF Ng was positively correlated with brain Aβ [37,82], CSF tau [22,38,47,48,83,84], and negatively with CSF Aβ [20,21,85]. The difference between Ng and T-tau was dependent on the level of Aβ in each clinical group [85]. In brain tissue from patients with familial and sporadic Alzheimer’s disease, Ng was significantly associated with the degree of amyloid and tau pathology [86]. SNAP-25 increased significantly with Aβ in the Aβ+ of MCI and AD when compared with Aβ- groups [17], was negatively correlated with CSF Aβ [49], and positively with CSF tau [49] and Ng [22]. Higher levels of SNAP-25–syntaxin interaction (controlling for the level of SNAP-25 as well as for pathology) were associated with a lower likelihood of dementia. An association between SNARE complex formation and cognitive function is in part regionally specific and may be more prominent for specific cognitive domains [85]. All synaptic biomarkers, Ng, SNAP-25, and synaptotagmin-1, were significantly positively correlated with CSF total tau concentrations in the AD biomarker group and higher concentrations appear to be related to AD pathology [22]. Postmortem assessments that showed lower brain complexin-I and -II levels contributed to cognitive dysfunction [53]. However not all are dependent on Aβ or Tau, and neuronal pentraxin 2(NPTX2) predicts progression in AD beyond Aβ1-42 and Tau [19]. 

Brain YKL-40 was co-localized with the astroglial marker GFAP but not with neuronal or microglial markers. In the brain, YKL-40 is expressed by a subset of astrocytes in AD and other tauopathies [53]. CSF YKL-40 was positively associated with T-Tau [48,50,54] and negatively with CSF Aβ42 [54,82], while no relationship was found with CSF Ng [83]. One study showed CSF YKL-40 to be correlated positively with CSF Aβ40 [55].

For the association of other inflammation with Aβ, microglial activation is positively associated with tau aggregation in MCI and AD while negatively associated with amyloid deposition [43]. Levels of Aβ and inflammation (11C-(R)-PK11195) can be seen to be related in MCI. However, the association between cortical tau tangles and inflammation in high amyloid-β cases was not detected [37].

NfL was negatively associated with CSF Aβ [83] and positively with CSF tau [56,83], CSF Ng [56,83], YKL-40 [47,56]. NfL reflects neurodegeneration independently of Aβ pathology [83]. Jin [56] reported that CSF NfL levels correlated with total tau, phosphorylated tau, and neurogranin but not with beta amyloid (Aβ). Multiple variables were differentially associated with CSF NfL and T-tau levels, but not Ng. Most associations were attenuated after adjustment for age and sex. T-tau had the strongest association with cognition in the presence of amyloidosis; followed by Ng. Variables associations with NfL did not differ by amyloid status [56].

Elevated VILIP-1 in AD was negatively associated with CSF Aβ [53,76] and positively with CSF tau [37], CSF Ng [84], and YKL-40 [53]. VILIP-1 increased significantly with Aβ in the Aβ+ of MCI and AD when compared with Aβ- groups [17].

Although reported to be associated with AD pathogenesis dependent on Aβ burden, some vascular risk factors of the *APOE ε4* genotype, higher systolic blood pressure etc. did not substantially increase the predictive performance in MCI with normal CSF Aβ; Aβ42 individually and in combination with t-tau and p-tau improved the prediction of progression to dementia [87]. In subjects without cognitive impairment, associations of vascular risk factors of cholesterol level, low-density lipoprotein cholesterol level, etc. with Aβ burden were found only among individuals who were not using vascular medications, which indicated that vascular medications can modulate the vascular risk of AD [88].

**Figure 2:**
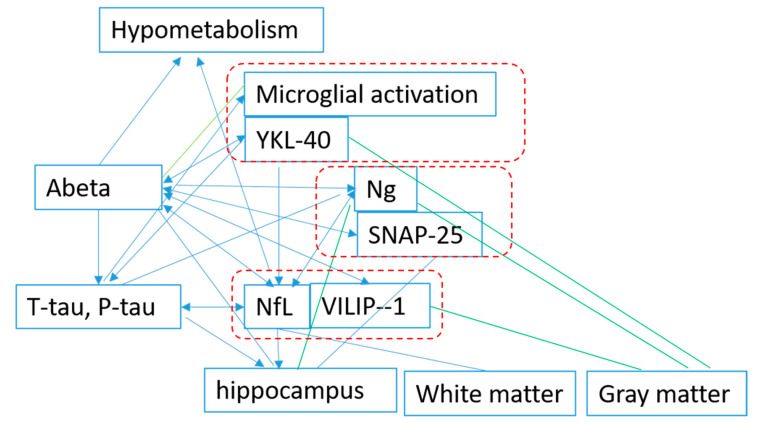
Association among the proteins based on studies published. Aβ: beta amyloid; Ng: neurogranin; SNAP-25: synaptosomal-associated protein 25; YKL-40: chitinase-3-like protein 1; NfL: neurofilament light chain; VILIP-1: visinin-like protein 1; Blue: association; Green: negative association.

Although there is inconsistency in the results, the majority of the results indicated that for biomarkers of t-Tau, P-tau, VILIP-1, SNAP-25, Ng, NfL, YKL-40, and inflammation (11C-(R)-PK11195), microglial activation began to change after the deposition of Aβ. NfL is also reported to be correlated with total tau independent of Aβ. The data on the association among other biomarkers is still limited, and it is difficult to identify interactions between biomarkers, except for Aβ (Figure 2).

### 3.2. Association of the Proteins with the Neuroimaging Findings and Phenotypes

We know that change in the brain structure can be measured by MRI metabolism using FDG-PET after the deposition of Aβ in brain. However, when and how the biomarkers are associated with the neuroimaging findings will be discussed in the next few paragraphs.

Brain Aβ is negatively associated with global or regional brain volume and cerebral metabolism measured by FDG-PET [31,89]. In amyloid-β-positive individuals, the atrophy across the entire brain is correlated with a summary measure of medial temporal lobe (MTL) 18F-AV-1451 uptake [33]. Aβ aggregation within the brain’s default mode network leads to regional hypo-metabolism, and an interaction between this hypo-metabolism and overlapping Aβ aggregation is associated with subsequent cognitive decline [44]. CSF Aβ is positive with global or regional brain volume [33] and cerebral metabolism measured by FDG-PET [51,52].

CSF tau is negatively associated with global or regional brain volume [46] and cerebral metabolism measured by FDG-PET [40,90]. In individuals without symptoms (Clinical Dementia Rating score, 0), the rates of change of CSF tTau and pTau181 are negatively associated with brain atrophy (high rates of change in CSF measures are associated with low rates of change in brain volume in asymptomatic stages). After symptom onset (Clinical Dementia Rating score, >0), an increased rate of brain atrophy id not associated with rates of change of levels of both CSF tTau and pTau181 [41]. Increased CSF sTREM2 is associated with accelerated cortical and hippocampal atrophy in cognitively unimpaired older participants, particularly in individuals with tau pathology [91]. Highly elevated CSF t-tau levels could indicate more cortical involvement presenting with early non-amnestic symptoms in atypical AD subtypes [57].

For the synaptic biomarkers, in the temporal lobe, the trimeric SNARE protein interaction (SNAP-25, syntaxin, VAMP) is associated with the rate of cognitive decline and global cognitive function [27]. SNAP-25 genotypes also correlate with a significantly decreased brain activity in the cingulate cortex and in the frontal and temporo-parietal area [92]. An elevated SNAP-25/Aβ42 ratio or higher Ng/BACE1 ratio is associated with the rate of hippocampal atrophy in pMCI and the rate of change of cognitive impairment in CN over the follow-up period [23,84,93]. One study also showed an association between longitudinal decline in white matter microstructure and change in Aβ42, phosphorylated-tau, total-tau, NFL, and Ng [94]. Elevated CSF Ng is associated brain atrophy [84,95]. Sometimes, studies have indicated the association of Ng with regional brain atrophy dependent on individual Aβ pathology. Ng is strongly associated with Aβ pathology, whereas NfL is more unspecific [96]. Portelius showed that Ng was not related with a change of brain volume or cerebral glucose metabolism (CGM) [20]. CSF Ng is associated with the rate of decrease in cortical glucose metabolism in the Alzheimer’s disease dementia group [20].

The levels of individual CSF biomarkers BACE1, Aβ1-40, Aβ1-38, and YKL-40 are all inversely correlated with the volume of gray matter of the precuneus in cognitively intact older subjects [31]. VILIP-1 is negatively associated with brain volume [52,53,97].

In inflammation, greater MCP-1, lower Aβ42, and greater P-Tau181 is associated with altered microstructure in the bilateral frontal, right temporal lobe, and microstructure in the precuneus, respectively [83]. Increased YKL-40 is associated with brain atrophy [20,50,51,53,83,98]. There is a strong correlation between YKL-40 and markers of neurodegeneration (total tau and p-tau), as well as a negative correlation between YKL-40 and cortical thickness (CTh) in AD-vulnerable areas in Aβ42+ subjects [51].

Plasma and CSF NfL is cognitive in MCI [39,99,100,101] and negatively associated with brain atrophy [39,99], hypometabolism [20,99,102], injury [98]. Serum NfL is not associated with amyloid-β deposition or glucose metabolism [99]. Elevated NfL predicts white matter damage in cognitively impaired older adults who are amyloid-negative and tau-positive [103]. Preische showed no relationship between CSF NfL and hypometabolism [39].

## 4. The Time Order of the Changes of the Biomarkers

Cerebrospinal fluid ratios with Aβ42 predict preclinical brain β-amyloid accumulation [104]. Studies have demonstrated that an increase in CSF tau and p-tau is a specific sign of AD progression that occurs downstream of the deposition of Aβ [105] and CSF p-tau was a significant predictor for PET-amyloid in SCD (subjective cognitive decline), MCI, and dementia [106].The time order of some biomarker changes is as follows CSF Aβ→CSF P-tau→CSF T-tau→PET Aβ−→PET tau. However, we have no knowledge of when the changes occur for other biomarkers and the timing with the MRI findings and glucose metabolism.

Only after Aβ PET became abnormal were the biomarkers of neuroinflammation, synaptic dysfunction, and neurodegeneration altered. Many findings provide in vivo support of the amyloid cascade hypotheses in humans [6]. Brain amyloid deposition quickly causes early changes in CSF tTau, pTau, SNAP-25, VILIP-1, and YKL-40. At the same time, the change of such factors also indicates performance on a cognitive composite, brain Aβ, 15–19 years before the estimated years from the onset of symptoms [102], which implies the interaction between biomarkers CSF SNAP-25, VILIP-1, and YKL-40 and Aβ42. Aβ42 also causes cortex atrophy [17]. CSF-amyloid is more sensitive early in the course of the disease. 

However, do the biomarkers change before or after the alteration of tau proteins? One study indicated that levels of inflammation could be seen in Aβ-positive MCI cases where 18F-flortaucipir signals were low, suggesting that microglial activation precedes tau tangle formation [82]. Some biomarkers changed before the accumulation of tau protein.

The change of NfL appears before the change in structural MRI findings and glucose metabolism measured by FDG-PET. In Aβ+ participants, NfL is associated with hypo-metabolism in AD-vulnerable regions [107]. Merluzzi suggests that NfL may be more sensitive to subclinical cognitive decline compared to other proposed biomarkers of neurodegeneration, whereas neurogranin and t-tau are not [108].The rate of change of serum NfL can distinguish mutation carriers from non-mutation carriers almost a decade earlier than cross-sectional absolute NfL levels (16.2 versus 6.8 years before the estimated onset of AD symptom) [41] and is correlated with EYO (estimated year of onset) and multiple cognitive and imaging measures [109].

Sometimes the biomarker change is not always associated with Aβ. Increased concentrations of baseline plasma t-tau predict structural basal forebrain cholinergic system (BFCS) atrophy progression in older adults at risk of AD, independently of β-amyloid status and APOE genotype [110]. Elevated CSF NfL levels but not CSF T-tau, P-tau, or neurogranin are risk factors for MCI in a community population, and they are independent of brain amyloid [23]. The rate of change of serum NfL is more associated with cortical thinning but less with amyloid-β deposition or glucose metabolism assessed by positron emission tomography [100]. Synapse dysfunction (i.e., dysplasticity) may be initiated early and is relatively independent of neuropathology-driven synapse loss [111].

In an animal model of AD, Ng levels increased in CSF when neurodegeneration was induced, peaking after 2 weeks, while it decreased in a brain when CSF Ng was a biomarker of synaptic degeneration [21]. CSF Ng levels are correlated with brain structure atrophy of hippocampal volumes, entorhinal volumes, and parahippocampal volumes in AD and with amyloid load in preclinical AD [112]. High baseline cerebrospinal fluid neurogranin levels in a mild cognitive impairment group were correlated with longitudinal reductions in cortical glucose metabolism and hippocampal volume at clinical follow-up [20]. CSF levels of synaptic and neuronal integrity biomarkers, amyloidogenic processing, and YKL-40 is associated with loss of structural integrity of brain regions implicated in the earliest stages of AD [95]. Baseline CSF VILIP-1 levels predict whole-brain, hippocampal, and entorhinal atrophy rates at least as well as tau and p-tau181 in early AD. Cognitively normal controls whose CSF VILIP-1, tau, or p-tau181 levels were in the upper tercile had higher rates of whole-brain, hippocampal, and entorhinal atrophy [97]. Almost all factors of neuroinflammation, synaptic function, neuronal injury, and neuronal degeneration experience changes prior to changes of the MRI findings and metabolism. A reduction in posterior cingulate glucose metabolism preceding a reduction in hippocampal volume has been observed [113]. What we know from the literatures is that the biomarkers experience changes prior to changes in metabolism, and then MRI findings follow the alternations of Abeta and Tau proteins, as shown in Figure 1. However, determining how long it takes from the change in biomarkers to the change in brain metabolism and structure, and the key biomarker pathway, requires further research. 

The information on the relationships among the biomarkers, particularly the time order of the change, is limited, such that the hypothesis on the pathogenesis of AD sounds imagined. More research on this topic is necessary.

## 5. Conclusions

We observed that the deposition of Aβ results in a change in the biomarkers related to synaptic function, neuroinflammation, and neural injury, in which NfL and Ng are majorly alternating. The relationship with tau protein is not consistent. Some research has shown that alternation occurs before the tau protein; but the relationship and time order of such biomarkers with tau proteins is not clear. The biomarkers experience changes prior to changes in metabolism and MRI findings. The Aβ cascade could be the main hypothesis; however, not all subjects converted to AD, even with very high elevated Aβ. The interaction between biomarkers and the time order of the change requires further research to identify the right subjects and right molecular target for precision medicine therapies. 

## Figures and Tables

**Table 1:** Association among the biomarkers at baseline.

T		AD	Brain Aβ	CSF Aβ	CSF Tau	CSF Ng	CSF SNAP-25	YKL-40	NF-L	VILIP-1	Brain Vol.	CGM
Beta amyloid	Brain Aβ	Increase	-									
	CSF Aβ	Decrease	Ne	-								
Tau	CSF Tau	Increase [14]	P [25]	Ne [16,26,27]	-							
Synaptic Function	CSF Ng	Increase [15]	P [25,28]	Ne [16,26,29]	P [18,25,28,29,30]	-						
	CSF SNAP-25	Increase [15,31]	NA	Ne [32]	P [32]	P [18]	-					
Neuro-inflammation	YKL-40	Increase [14]	NA	Ne [25,33]	P [34,35,36]	No [37]	NA	-				
Neuro-injury	NfL	Increase [14]	No [38,39]	Ne [29,40], No [41]	P [29,38,40]	P [42,43,44]	NA	Positve [34,38]	-			
	VILIP-1	Increase [45]	NA	Ne [35,45]	P [35]	P [46]	NA	P [35]	NA	-		
Brain structure	Brain vol.	Decease	N [47,48]	P [49]	Ne [50]	Ne [34,49] No [16]	Ne [33]	Ne [35,36,49]	Ne [16,42,44,51]	Ne [33,36,52]	-	
hypometabolism	CGM	Decrease	Ne [47,48]	P [53,54]	Ne [55,56]	Ne [16]	NA	NA	Ne [16,29,40,57], No [41]	NA	P	-

P: positive; Ne: negative; No: no relationship; NA: not available; CGM: cerebral glucose metabolism; CSF: cerebral spinal fluid; Aβ: beta amyloid; Ng: neurogranin; SNAP-25: synaptosomal-associated protein 25; YKL-40: chitinase-3-like protein 1; NfL: Neurofilament light chain; VILIP-1: visinin-like protein 1; [] reference number.

## Abbreviations

hFABp	heart-type fatty acid-binding protein
CAA	cerebral amyloid angiopathy
CBF	cerebral blood flow
CGM	cerebral glucose metabolism
FDG-PET	18F-positron emission tomography-computed tomography
CSF	cerebral spinal fluid
CSF Aβ	cerebral spinal fluid beta amyloid
CSF Tau	cerebral spinal fluid Tau protein
PET-Aβ	amyloid-beta assessed by florbetapir PET
Ng	neurogranin
SNAP-25	synaptosomal-associated protein 25
YKL-40	chitinase-3-like protein 1
NfL	neurofilament light chain
VILIP-1	visinin-like protein 1
BACE	beta-site APP cleaving enzyme 1
SUVR	standardized uptake value ratio
MCI	mild cognitive impairment
CN	cognitive normal
FTLTD	frontotemporal lobar degeneration (explained in line 140)
Iba 1	ionized calcium-binding adapter molecule
GFAP	fibrillary acidic protein
TREM-2	triggering receptor expressed on myeloid cells 2
